# Bilateral Recurrent Laryngeal Nerve Injury Following Botulinum Toxin Injection at the Cricopharyngeus Muscle: Diagnosis, Anatomic Considerations, and ICU Management

**DOI:** 10.7759/cureus.68798

**Published:** 2024-09-06

**Authors:** Mark A Potesta, Akram Shibani

**Affiliations:** 1 Medical School, Lake Erie College of Osteopathic Medicine, Bradenton, USA; 2 Pulmonary and Critical Care, Ascension St. Vincent Hospital, Jacksonville, USA

**Keywords:** bilateral recurrent laryngeal nerve, bilateral vocal cord paralysis, bilateral vocal cord paresis, bl rln palsy, bl rln paralysis, botulinum cricopharyngeus, botulinum risk, cricopharyngeus, cricophayngeus muscle injury, recurrent laryngeal nerve palsy

## Abstract

Bilateral vocal cord paralysis poses life-threatening risks to patients who do not undergo prompt diagnostic intervention and airway management. Although developing bilateral vocal cord paralysis is extremely rare, if injury does occur, it is more frequently due to surgical resection sequelae in the neck. This case is particularly unique as we present a patient with a history of stage III laryngeal carcinoma status post chemotherapy in remission, who developed respiratory distress three days following an upper endoscopy procedure for an esophageal stricture at the level of the cricopharyngeus muscle, where he received a botulinum injection. This manuscript discusses the anatomy, clinical practices of botulinum toxin, nerve innervation, and mechanisms of injury for patients who develop bilateral recurrent laryngeal nerve injury. In addition, this manuscript details vocal cord positioning and how the positioning of the cords during laryngoscopy investigation can lead to diagnostic confirmation. With few reported cases of bilateral recurrent laryngeal nerve injury secondary to botulinum toxin particularly at the cricopharyngeus level, this report should serve as a guide for future clinicians regarding the risks of using this toxin, the risks of local spread, and management.

## Introduction

Vocal cord paralysis is exceedingly rare and poses a life-threatening risk to patients. The vocal cords serve two pertinent purposes, which are phonation and protection of the lower airways from aspiration via glottic patency [1]. In addition, there are various etiologies of paralysis with subsequent vocal cord positioning and symptomatology. For example, if the vocal cords are bilaterally paralyzed in a more medial position, stridor and respiratory distress are likely to be the predominant clinical manifestation [1]. Bilateral paralysis in a more medial position is also less likely to cause significant change in phonation or cause significant aspiration [1]. Alternatively, if both cords are paralyzed in a more lateral position, the airway will remain open, increasing the risk of aspiration of choking events [1]. The lateral position is also far less likely to present with stridor and respiratory distress [1]. The recurrent laryngeal nerve (RLN) innervates motor supply to the intrinsic laryngeal muscles (abductors), while also providing sensory supply to mucosa below the vocal cords [2]. Damage to both recurrent laryngeal nerves leading to bilateral vocal cord paralysis is the most common form of injury [3]. Classically, this type of injury is seen as related to surgery during thyroid resection, tracheal resection, esophageal resection, or as a result of prolonged intubation [3]. The incidence of this type of bilateral injury is exceedingly rare (estimated at 0.2% of thyroid surgical procedures) [2]. Of the reported cases of bilateral vocal cord paralysis, studies have estimated injury can be attributed to surgical trauma in ~44% of cases, malignancies in ~17% of cases, secondary to intubation in ~15% of cases, and ~12% due to neurological and idiopathic causes [3].

## Case presentation

**Figure 1:**
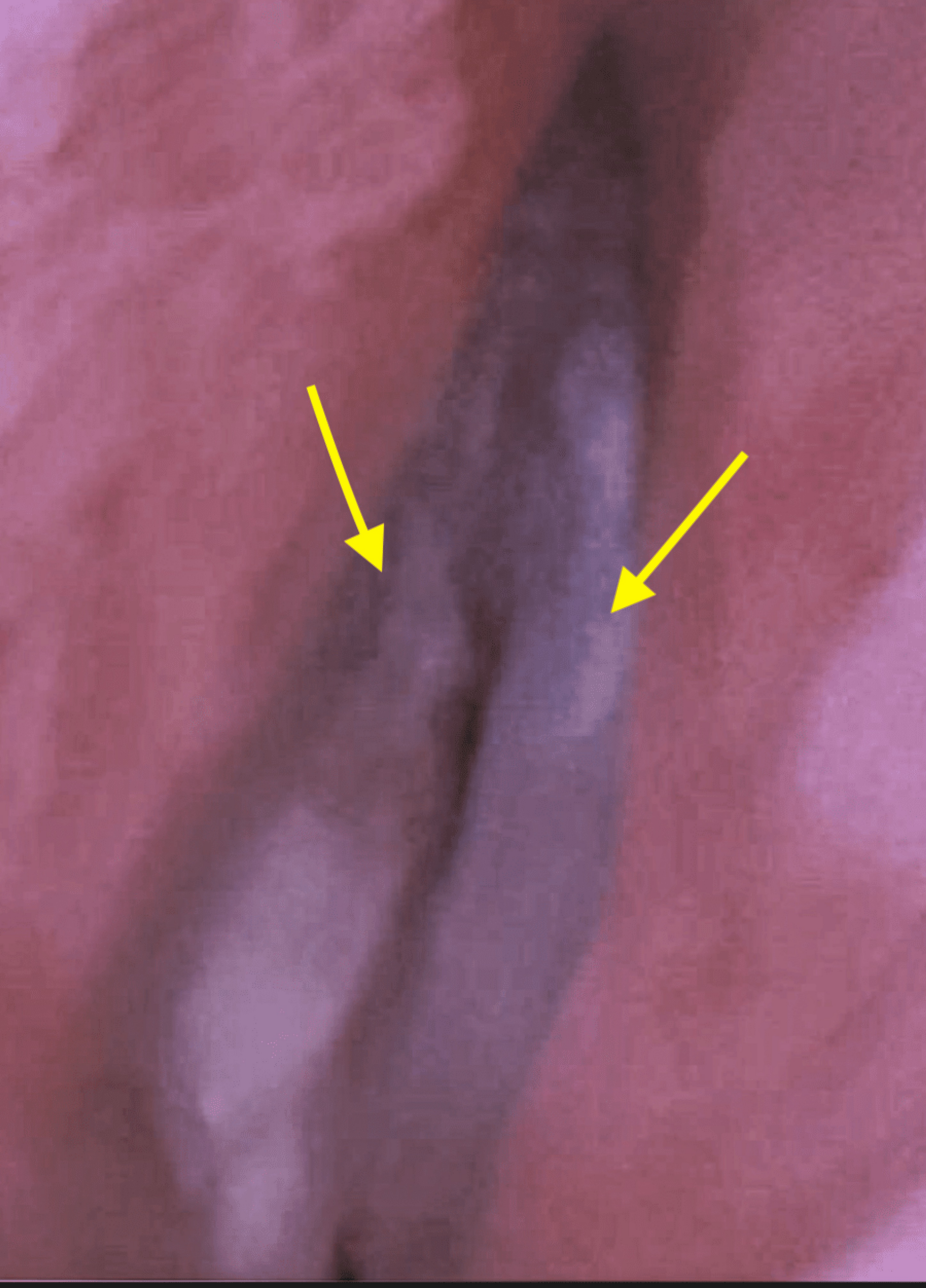
Patient’s vocal cords paralyzed in a median position during inhalation, exhalation, quiet respiration, and phonation, confirmed on laryngoscopy.

A 66-year-old Caucasian male with a past medical history of GERD and stage III laryngeal carcinoma status post chemotherapy in remission presented to the emergency department with shortness of breath, cough, and wheezing. The patient noted the symptoms began three days ago, following an upper endoscopy procedure for an esophageal stricture at the level of the cricopharyngeus muscle, where he received a botulinum injection of 100 units. In particular, the patient noted worsening symptoms of dysphonia, shortness of breath, and difficulty swallowing over the course of the three days. On exam, he had diminished breath sounds bilaterally, a loud inspiratory stridor, and was having difficulty protecting his airway. He was afebrile and had a SpO_2_ of 90% on 2 L of O_2_. The patient was subsequently admitted to the intensive care unit and underwent fiberoptic laryngoscopy/bronchoscopy, which was suggestive of critical airway with bilateral vocal cord paralysis. Diffuse laryngeal edema and erythema were noted, true vocal cords were examined, and both vocal cords were in the median position. The patient was asked to inhale and phonate, which did not produce mobility of the vocal cords, confirming the diagnosis (Figure 1). The patient was sedated and status-post nasal intubation and mechanically ventilated for airway protection. His labs were unremarkable (Table 1). He was started on corticosteroids for laryngeal edema, heparin SQ for deep vein thrombosis (DVT) prophylaxis, and protonix for peptic ulcer disease prophylaxis. ICU management of the patient’s airway remained the priority as the discussion of a trial of pyridostigmine versus tracheostomy remained a topic of discussion.

**Table 1:** Lab results

Test	Result	Reference range
White blood cells (WBC)	5,500 cells/µL	4,500-11,000 cells/µL
Red blood cells (RBC)	5.2 million cells/µL	4.7-6.1 million cells/µL
Hemoglobin	14.1 g/dL	12.6-16.7 g/dL
Hematocrit	42%	36.9-48.5%
Platelets	249,000 cells/µL	150,000-450,000 cells/µL
Glucose	89 mg/dL	70-100 mg/dL
Potassium	4.2 mmol/L	3.5-5.1 mmol/L
Blood urea nitrogen (BUN)	14 mg/dL	7-20 mg/dL
Creatinine	0.9 mg/dL	0.6-1.2 mg/dL

## Discussion

**Figure 2:**
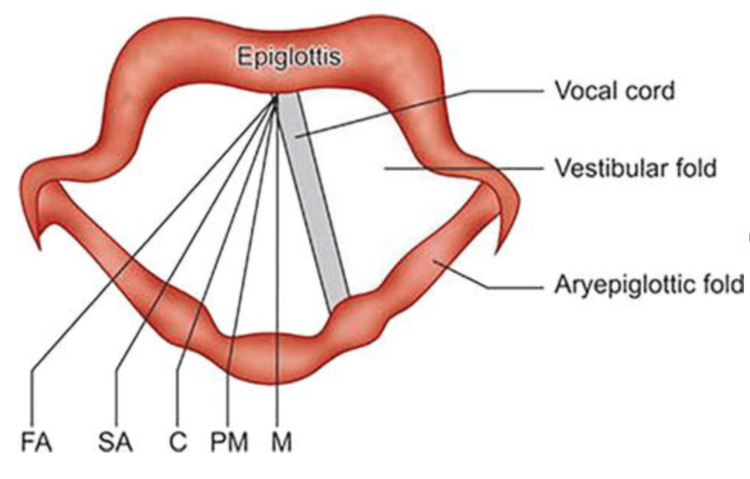
Positions of the vocal cords M: median position (midline), PM: paramedian (1.5 mm from the midline), C: cadaveric (3 mm from the midline/neutral position), SA: slight abduction (7 mm from the midline), FA: full abduction (9 mm away from the midline) Source: Priyamvada, 2022 [4] Permission is obtained from the original publisher under Creative Commons license [4].‌

There are a variety of causes of bilateral vocal cord paralysis, such as scarring, iatrogenic, malignancy, CNS pathology related, or systemic disease related [1]. These general categories can further be broken down into a subset of causes with different mechanisms of pathologic insult. Scarring can be secondary to prolonged intubation, inhalation of toxins, inflammatory disease, or radiation treatment [1]. These conditions can cause severe fibrosis of the vocal cords, despite the muscles “functioning” [1]. Iatrogenic damage to the recurrent laryngeal nerve often occurs following surgical intervention to the thyroid, trachea, or esophagus [1]. Bilateral recurrent laryngeal nerve injury implicates both abduction and adduction mechanisms of the vocal cords, as there is a role in the innervation of both types of muscle fibers [1]. Although both types of muscle fibers have innervation attributed to the recurrent laryngeal nerve, the adductor muscle fibers are four times greater than the abductor muscle fibers [1]. As a result of this difference in magnitude in innervation, the adductor muscle fibers are affected much more significantly during injury, paralyzing the vocal cords in a paramedian position, such as seen in the patient in this case (Figure 2) [1]. Malignancy is also a possible mechanism of injury as tumor bulk can lead to paralysis by impairing the normal motion of vocal cords [1]. Neurological causes, such as multiple sclerosis, Guillain-Barre syndrome, or amyotrophic lateral sclerosis (ALS), can also implicate the nuclei of the vagus nerves, which are directly responsible for the innervation of the recurrent laryngeal nerves [1].

Understanding the anatomy of the muscles involving the larynx and pharynx and their relative innervations is essential for deciphering how iatrogenic injury can occur. As previously stated, most iatrogenic injuries occur following thyroid surgery, esophageal resection, or tracheal resection. This case we are presenting is particularly rare as paralysis occurred secondary to botulinum toxin for treating a stricture at the level of the cricopharyngeus muscle, not involving resection. The major component of the upper esophageal sphincter is the cricopharyngeal muscle, which is part of the inferior constrictor muscles [5]. The cricopharyngeal muscle is chiefly involved in separating the pharynx from the esophagus, serving as a high-pressure zone, and maintaining the upper esophageal sphincter’s closure at rest [5]. Motor innervation of the cricopharyngeal muscle has been debated over time; however, a 2016 study has provided evidence that the recurrent laryngeal nerve, when stimulated, evoked a motor response in 90% of cricopharyngeal muscles, with the external branch of the superior laryngeal nerve contributing the remainder [5]. This is particularly significant as the recurrent laryngeal nerve also serves as the main source of innervation to the adductor muscles of the vocal cords, with damage to the nerves bilateral causing the cords to assume a median or paramedian position [1]. 

Botulinum toxin works by blocking the release of acetylcholine, causing neuromuscular blockade and subsequent paralysis [6]. Botulinum toxin has been FDA-approved for a variety of medical conditions such as chronic migraine, cervical dystonia, blepharospasm, axillary hyperhidrosis, bladder dysfunction, spasticity, and cosmetic use [7]. In addition, botulinum has been used to treat esophageal strictures and prevent collagen deposition and subsequent fibrosis [8]. Although botulinum toxin is generally safe, it does pose the risk of unforeseen consequences. The local and systemic distributions of botulinum toxin depend on spread, diffusion, migration, volume, and distribution [6]. Spread refers to from one site to another, based on physical factors, whereas diffusion refers to a microscopic passive transport of a soluble molecule beyond the original site of botulinum injection [6]. Migration refers to the spread of distant sites that can occur by nerves or blood [6]. It is through these mechanisms, predominantly spread, diffusion, and migration, that botulinum in this case is able to affect both recurrent laryngeal nerves.

Understanding the position of the vocal cords in healthy and diseased states is also vital for the confirmation of vocal cord paralysis during laryngoscopy. In a healthy state, the vocal cords assume a median position during phonation, are aligned in a paramedian position during a strong whisper, in the cadaveric (neutral) position at rest, and are aligned in gentle abduction during quiet respiration and full abduction during deep respiration [4]. In a diseased state, the cords assume a median or paramedian position with recurrent laryngeal nerve paralysis, with the positions of the cords not changing during the breath cycle (Figures 1, 2) [4].

The debate surrounding the ICU management of this patient focused on whether intervention should be warranted with the use of botulinum antitoxin versus pyridostigmine (acetylcholinesterase inhibitor) versus tracheostomy. Airway management took precedence, nasal intubation, and mechanical ventilation following laryngoscopy. Ultimately, the patient underwent a tracheostomy for the suspected need for long-term airway management. Botulinum antitoxin was deemed to be inappropriate in this case due to the paralysis having already occurred [9]. The patient was also treated with an IV steroid regimen during his week-long ICU stay for his associated laryngeal edema. He exhibited marked improvement to this treatment, was successfully weaned off the ventilator, and was not in respiratory distress with a tracheostomy in place. In addition, the patient was monitored for systemic botulinum disease manifestation (i.e., systemic descending paralysis) during the remainder of his ICU stay; however, it remained localized to his vocal cords. There is evidence to suggest that pyridostigmine has a role in the reversal of severe botulinum toxicity, but due to the patient’s improvement with tracheostomy and localized involvement, the risk of potential systemic adverse cholinergic effects with pyridostigmine was deferred inpatient [10].

## Conclusions

The patient was subsequently discharged with his tracheostomy tube in place, not in respiratory distress. It is unclear at this time whether his paralyzed vocal cords will spontaneously reverse in the interim, or if they are more likely to take a more indolent course to recovery that is consistent with a typical three- to six-month timeline of botulinum injection. Regardless, the patient's progress will be followed outpatient, with a plan to perform a subsequent laryngoscopy at a later date. This case highlights the standalone rarity of developing a bilateral recurrent laryngeal nerve palsy coupled with an atypical mechanism of injury. Furthermore, this case emphasizes the importance of understanding anatomy, nerve innervation, and treatment. Future uses of botulinum toxin should be exercised with caution and clinicians should be cognizant of the potential risks for bilateral vocal cord paralysis when treating esophageal strictures, particularly at the level of the cricopharyngeus muscle.
